# The Proposed Copyright Law

**Published:** 1883-06

**Authors:** 


### THE PROPOSED COPYRIGHT LAW, AND ITS RELATION TO MEDICAL AUTHORSHIP.

BY "MEDICUS AMERICANUS."

The question of the relations of American and foreign authors and publishers has again appeared in Congress. Some American authors, headed by Dr. Oliver Wendell Holmes, have petitioned Congress on the subject of the duty upon books printed in foreign countries ; and the fact is stated that foreign publishers may reprint American works and, without regard to copyright, and in consequence of the cheapness of foreign labor and materials, may send those works back to this country and put them in the market at prices which will exclude the original publications, and deprive the authors of the royalty upon their works.

The foreign publishers excuse themselves for this injustice to American authors, by retorting that American publishers are equally unjust to the foreign authors in publishing their works without compensation, in the absence of an international copyright law.

That there is ground for complaint on the part of authors, both American and foreign, for this state of things, there can be no question. To the American authors of literary works, whether profes-fessional or not, there is a double wrong, or at least a double disadvantage. They suffer from the competition of the publication of similar foreign works in this country at less prices, because nothing is paid the authors, unless it be a pittance as a gratuity, and they are liable to have their works reprinted abroad and sold in this country, crowding the original copyrighted works out of the market.

It is, however, claimed that this acknowledged injustice to authors is compensated for (as if anything could compensate for injustice) by the advantage to the public in furnishing cheap books. This alleged advantage to our profession, besides the matter of injustice to a portion of them, is not without very serious drawbacks.

In the first place, by this state of things original research and the production of works requiring elaborate preparation are discouraged among us, and our general professional advancement is retarded.

But a much more serious evil arises from the fact that, by the multiplication of foreign works, particularly upon medical diseases, displacing those of original, home production, the profession is educated in views of *treatment* not adapted to the peculiarities of the ordinary diseases of this country, especially those prevailing in the rural districts.

While the facts and principles of physical, chemical, physiological, and to a large extent, pathological science are the same everywhere, all know that particular diseases and their indications for treatment are greatly modified by the previous history, the surroundings, and the peculiar habits and conditions of patients.

The mechanical principles of surgery and obstetrics are in all places the same; but even in surgical and obstetrical practice the general conditions of the patients will often materially influence the course to be pursued, and the results of mechanical measures, and especially the constitutional treatment, will require to be modified by the state and surroundings of the individuals.

It is too obvious to need more than the simple statement that conditions of patients in Europe differ from those in this country, and that there is an immense difference between the poor, starved, intemperate or vicious persons found in the general hospitals in large European cities, and the masses of temperate and well-to-do people in this country. Climate, locality, habits—everything—are vastly different.

It must be admitted that those physicians who have derived their experience and drawn their conclusions from European city hospital practice, supplemented only by a consultation and office practice—which is the case with nearly all our foreign authors—very seldom seeing acute diseases in any other than their severer forms, and in their advanced stages—cannot be considered as the best, or, indeed, as safe guides for those engaged in general practice, as those who see patients in the beginning of their diseases, whether in cities, villages or the rural districts of our country.

It is much to be feared that the almost exclusive reading of foreign works, written by men with the kind of experience indicated, has led, and is leading, to wrong views of the adaptability and powers of therapeutical agents, and is in the greatest danger of leading to inefficient and positively injurious modes of treatment.

Extremes in our profession as elsewhere are to be met with, and while over confidence in medicines not unfrequently exists, leading to excessive medication, a want of confidence in therapeutical agents, or medical nihilism, is quite as great an evil; and this is likely to be formed, and to be fostered and communicated by those whose practice has been in European city hospitals, and in consultations in the later stages of extreme cases.

We do not wish to be understood as objecting to the publication and the reading of foreign works on the practice of medicine ; but we insist that too much dependence upon and too closely following such works in general medical practice is often disastrous to the interests of our patients. We fear that some of our American writers on practical medicine are too much influenced by foreign writers, whose experiences have been so different from the general practitioners among us.

We are glad to see that signs of change from this state of things are appearing. In Prof. Flint's recent productions more attention is given to therapeutics, and a little more confidence seems to be entertained in the power of remedies. Prof. Bartholow's writings mark a period of reaction from medical negation ; and the still more recent work of Prof. Palmer, which is the result of his long experience as a practitioner and teacher, and of extended observation in this and other countries, and which, while giving evidence of study, is written from an American stand-point, is clear in its statements and reasonable in its conclusions, and is calculated to inspire confidence in the power of remedies, especially when applied in the early stages of disease, is yet more encouraging.

An examination of this work has suggested some of the foregoing thoughts which we deem important to be brought to the minds of the profession in these times of abounding foreign publications, and when the interests of some publishers and controllers of medical journals inspire laudationsofthe re-published foreign works, and unfair and even untruthful statements in disparagement of American productions.

While giving heed to the results of investigation and experience from every quarter, we want American practical works for American practicing physicians, and American therapeutics for the peculiarities of American diseases. We want for students and practitioners, pathological and therapeutical principles clearly stated, and explicit directions for the treatment of diseases in their incipiency, in their early as well as in their advanced stages, in their milder as well as in their severer forms, in the country as well as in the city, and in private and general as well as in hospital and special practice. We are glad to know that American works are being produced specially designed for meeting these requirements, and that this last production has been so successful in accomplishing its purpose.

